# Semiparametric estimation of the proportional rates model for recurrent events data with missing event category

**DOI:** 10.1177/09622802211023975

**Published:** 2021-06-18

**Authors:** Feng-Chang Lin, Jianwen Cai, Jason P Fine, Elisabeth P Dellon, Charles R Esther

**Affiliations:** 1 Department of Biostatistics, University of North Carolina at Chapel Hill, NC, USA; 2 Department of Medicine, University of North Carolina at Chapel Hill, NC, USA

**Keywords:** Cystic fibrosis, generalized partially linear model, polynomial spline, rate proportion, weighted estimating equation

## Abstract

Proportional rates models are frequently used for the analysis of recurrent event data with multiple event categories. When some of the event categories are missing, a conventional approach is to either exclude the missing data for a complete-case analysis or employ a parametric model for the missing event type. It is well known that the complete-case analysis is inconsistent when the missingness depends on covariates, and the parametric approach may incur bias when the model is misspecified. In this paper, we aim to provide a more robust approach using a rate proportion method for the imputation of missing event types. We show that the log-odds of the event type can be written as a semiparametric generalized linear model, facilitating a theoretically justified estimation framework. Comprehensive simulation studies were conducted demonstrating the improved performance of the semiparametric method over parametric procedures. Multiple types of *Pseudomonas aeruginosa* infections of young cystic fibrosis patients were analyzed to demonstrate the feasibility of our proposed approach.

## 1 Introduction

Recurrent event data with multiple categories frequently arise in medical science and population health studies. Different causes of hospitalizations, multiple strains of bacteria infections, and various types of treatment failures all belong to such data type. Taking cystic fibrosis (CF) as an example, recurrent *Pseudomonas aeruginosa* (PA) infections are commonly observed in patients with CF. PA infection includes mucoid and nonmucoid strains. Without appropriate treatment, the recurrent infections with mucoid strains often become persistent and chronic, causing increased CF mortality and morbidity.^1–3^ As another example, patients who received renal transplants may have different types of recurrent infections.^4^ End-stage renal disease patients who received continuous ambulatory peritoneal dialysis may have multiple types of treatment failures that make the patient switch to other dialysis methods.^5^

When modeling this kind of recurrent event data, a proportional rates model that is conditional only on the current value of covariates is commonly used.^6^ Let $N_{ij}^{*}(t)$ denote the number of recurrent events up to time *t* for subject *i* (i=1,…,n) and event category *j* (j=1,…,J). Let $Z_{ij}(t)$ denote the column vector of covariates which are possibly time-varying. A proportional rates model proposed by Cai and Schaubel^6^ is defined by

(1) $$E\{dN_{ij}^{*}(t)|Z_{ij}(t)\}=\exp\{\beta_{0}^{\text{T}}Z_{ij}(t)\}d\mu_{0j}(t)$$

where *β*_0_ is the regression coefficient and $d\mu_{0j}(t)$ is the baseline rate function for the *j*th type of the recurrent event. Although *β*_0_ is not indexed by event category *j*, the model is flexible enough to accommodate covariate effects that are specific to the event type in each individual model. For example, if there are two types of recurrent events and one uses *q*_1_- and *q*_2_-column vector of covariates, ${\tilde{Z}}_{i1}$ and ${\tilde{Z}}_{i2}$, for the first and second type of recurrent events, respectively, one can define $Z_{i1}={\left({\tilde{Z}}_{i1}^{\text{T}},0_{q_{2}}^{\text{T}}\right)}^{\text{T}}$ and $Z_{i2}={\left(0_{q_{1}}^{\text{T}},{\tilde{Z}}_{i2}^{\text{T}}\right)}^{\text{T}}$ to specify the individual models, where $0_{q_{j}}$ is a *q_j_*-column vector of zeros. Accordingly, one may define $\beta_{0}={\left(\beta_{1}^{\text{T}},\beta_{2}^{\text{T}}\right)}^{\text{T}}$, where $\beta_{j}={\left(\beta_{j1},\ldots ,\beta_{jq_{j}}\right)}^{\text{T}}$ for *j *=* *1, 2.

Let $Y_{i}(t)=I(C_{i}\geq t)$ indicate whether subject *i* with censoring time *C_i_* is under observation at time *t*, where t∈[0,τ], and *τ* is the end of follow-up time. Let $N_{ij}(t)=Y_{i}(t)N_{ij}^{*}(t)$ denote the observed number of events up to time *t*. When the event category is fully observed, Cai and Schaubel^6^ showed that the coefficient *β*_0_ in (1) could be consistently estimated by the estimating equations

(2) $$U^{n}(\beta )=\sum_{i=1}^{n}\sum_{j=1}^{J}\int_{0}^{\tau }\{Z_{ij}(t)-{\bar{Z}}_{j}(t;\beta )\}\;\text{d}N_{ij}(t)=0$$

where ${\bar{Z}}_{j}(t;\beta )=S_{j}^{\left(1\right)}(t;\beta )/S_{j}^{\left(0\right)}(t;\beta )$ with

$$S_{j}^{\left(d\right)}(t;\beta )=n^{-1}\sum_{i=1}^{n}Y_{i}(t)Z_{ij}{\left(t\right)}^{⊗d}\exp\{\beta^{\text{T}}Z_{ij}(t)\}$$

for *d *=* *0, 1, where $a^{⊗0}=1,\;a^{⊗1}=a$, and $a^{⊗2}=aa^{\text{T}}$ for a column vector *a*.

However, when the event category is possibly missing, the estimating equation (2) is not feasible since the quantity $dN_{ij}(t)$ is not always observable. A naive approach, which uses completely observed data that include only events with known type, can be valid if the event category is missing completely at random, but may give biased results if the missingness depends on the covariates. Schaubel and Cai^5^ suggested that one rewrite $dN_{ij}(t)$ as

(3) $$dN_{ij}(t)=R_{i}(t)dN_{ij}(t)+\{1-R_{i}(t)\}\delta_{ij}(t)dN_{i\cdot }(t)$$

where $R_{i}(t)$ indicates whether the event category is observed, $\delta_{ij}(t)$ indicates whether the event category is type *j*, and $dN_{i\cdot }(t)=\sum_{j=1}^{J}dN_{ij}(t)$ indicates the total number of events at time *t*. Note that $dN_{i\cdot }(t)$ equals 0 or 1 since they assume events with different types do not occur simultaneously. They further suggested that one replace the unknown quantity $\delta_{ij}(t)$ with a consistent estimator for $p_{ij}(t)$, where

$$p_{ij}(t)=E\{\delta_{ij}(t)|dN_{i\cdot }(t)=1,Z_{ij}(t)\}$$

One may consider parametric, multinomial logit models for estimation of $p_{ij}(t)$.^5,7^ However, the association between the covariates and *δ_ij_* may not be correctly specified, which may lead to inconsistent estimation. This motivates us to develop a more robust method that weakens the impact of model misspecification.

In this paper, we extend the proportional rates method previously developed for estimation of $p_{ij}(t)$^8^ using a semiparametric approach that exploits a special form of the rate proportion of event type *j* to the overall rate function. Interestingly, under the proportional rates model (1), the ratio of two rate proportions can be expressed as

(4) $$\log\{p_{ij}(t)/p_{iJ}(t)\}=\beta_{0}^{\text{T}}X_{ij}(t)+\eta_{0j}(t)$$

where $X_{ij}(t)=Z_{ij}(t)-Z_{iJ}(t)$ and $\eta_{0j}(t)=\log\{d\mu_{0j}(t)/d\mu_{0J}(t)\}$. In fact, model (4) can be viewed as a generalized partially linear model. Under certain regularity conditions, one can estimate *β*_0_ and $\eta_{0j}$ simultaneously via semiparametric regression techniques such as local polynomials,^9^ generalized additive models,^10^ and polynomial spline functions.^11^

When there is no missing data in the event type, one can estimate $p_{ij}(t)$ using all of the data based on model (4). With missing event types, however, one can only estimate $p_{ij}^{c}(t)$ using completely observed data, where

$$p_{ij}^{c}(t)=E\{\delta_{ij}(t)|dN_{i\cdot }(t)=1,R_{i}(t)=1,Z_{ij}(t)\}$$

Letting $\pi_{ij}(t|Z_{ij})=E\{R_{i}(t)|dN_{ij}(t)=1,Z_{ij}(t)\}$ denote the probability of non-missingness given that the event type *j* occurs, one can show

(5) $$\log\{p_{ij}^{c}(t)/p_{iJ}^{c}(t)\}=\beta_{0}^{\text{T}}X_{ij}(t)+\eta_{0j}(t)+\kappa_{j}(t|Z_{ij})$$

where $\kappa_{j}(t|Z_{ij})=\log\{\pi_{ij}(t|Z_{ij})/\pi_{iJ}(t|Z_{iJ})\}$ is the log-ratio of non-missingness for two event types. Since model (5) is time-varying, estimation is complicated. A common assumption in the previous literature is that $\pi_{ij}(t|Z_{ij})$ does not depend on *j* and $\kappa_{j}(t|Z_{ij})=0$ for each *j*.^5,7,8^ This assumption corresponds to missing at random (MAR) assumption when the missingness does not depend on unobserved information.^12^ In this paper, we adopt the same assumption and assume $\kappa_{j}(t|Z_{ij})=0$.

Theoretical challenge remains when the semiparametric estimator of $p_{ij}(t)$ is substituted for the unknown $\delta_{ij}(t)$ in the estimating equations for *β*. Specifically, it is not clear if one can still obtain a $n^{1/2}$ convergence rate in the estimation of *β* since the convergence rate of $p_{ij}(t)$ estimation is generally slower than $n^{1/2}$ with a semiparametric approach. We will show that, under mild regularity conditions, our estimator of *β* converges at a $n^{1/2}$ rate to a normal distribution with variance that may be consistently estimated using a simple plug-in formula.

The remaining sections are organized as follows. In Section 2, we exploit a cubic B-spline function for the estimation of $p_{ij}(t)$ and propose general estimating equations for the regression coefficient *β*_0_ and baseline mean function $\mu_{0j}(t)$ in model (1). Consistency and large sample normality of the estimators are shown in Section 3. Finite-sample performances evaluated by comprehensive simulation experiments are studied in Section 4. A real-data analysis on multiple types of PA infections in the United States 2016 Cystic Fibrosis Foundation Patient Registry is presented in Section 5. Conclusions and discussions on future research are presented in Section 6.

## 2 Estimation method

Assume that $\eta_{0j}(t)$ can be approximated by a cubic B-spline function

$${\tilde{\eta }}_{0j}(t;\xi_{j})=\xi_{j0}+\sum_{k=1}^{m+3}\xi_{jk}b_{k}(t)$$

where $b_{k}(t)$ (k=1,…,m+3) are basis functions, *m* is the number of interior knots, and $\xi_{j}={\left(\xi_{j0},\ldots ,\xi_{j(m+3)}\right)}^{\text{T}}$ is a vector of spline coefficients. Let $\theta_{0}={\left(\beta_{0}^{\text{T}},\xi_{0}^{\text{T}}\right)}^{\text{T}}$, where $\xi_{0}={\left(\xi_{1}^{\text{T}},\ldots ,\xi_{J-1}^{\text{T}}\right)}^{\text{T}}$. One can estimate *θ*_0_ by maximizing an approximate log-likelihood function

(6) $$\ell (\theta )=\sum_{i=1}^{n}\int_{0}^{\tau }\ell_{i}(t;\beta ,\xi )R_{i}(t)\;\text{d}N_{i\cdot }(t)$$

where

$$\ell_{i}(t;\beta ,\xi )=\sum_{j=1}^{J}\delta_{ij}(t)m_{ij}^{c}(t)-\log\left[\sum_{j=1}^{J}\exp\{m_{ij}^{c}(t)\}\right]$$

with $m_{ij}^{c}(t;\theta )=\beta^{\text{T}}X_{ij}(t)+\xi^{\text{T}}B_{j}(t)$, where $B_{j}(t)$ is a (m+4)×(J−1) column vector with $b(t)={\left(1,b_{1}(t),\ldots ,b_{m+3}(t)\right)}^{\text{T}}$ in the *j*th block for j=1,…,J−1, and $B_{J}(t)=0$ for all *t*. The number of interior knots *m* can be selected by Akaike information criteria (AIC) that minimizes −2ℓ(θ) plus two times the number of parameters in *θ*. However, other approaches such as generalized cross-validation that approximates the leave-one-out cross-validation may also be considered.^13^

Letting $\tilde{\theta }={\left({\tilde{\beta }}^{\text{T}},{\tilde{\xi }}^{\text{T}}\right)}^{\text{T}}$ denote the maximizer of (6), one can estimate $p_{ij}(t)$ by

$$p_{ij}(t;\tilde{\theta })=\frac{\exp\{{\tilde{\beta }}^{\text{T}}X_{ij}(t)+{\tilde{\xi }}^{\text{T}}B_{j}(t)\}}{\sum_{\ell =1}^{J}\exp\{{\tilde{\beta }}^{\text{T}}X_{i\ell }(t)+{\tilde{\xi }}^{\text{T}}B_{\ell }(t)\}}$$

By solving the estimating equations

(7) $$U^{r}(\beta )=\sum_{i=1}^{n}\sum_{j=1}^{J}\int_{0}^{\tau }\{Z_{ij}(t)-{\bar{Z}}_{j}(t;\beta )\}\text{d}N_{ij}^{r}(t;\tilde{\theta })=0$$

where $dN_{ij}^{r}(t;\theta )=R_{i}(t)dN_{ij}(t)+\{1-R_{i}(t)\}p_{ij}(t;\theta )dN_{i\cdot }(t)$, one can obtain our proposed estimator ${\hat{\beta }}^{r}$ for *β*_0_. With *β* replaced by ${\hat{\beta }}^{r}$ in the estimating equation

$$\sum_{i=1}^{n}\sum_{j=1}^{J}\int_{0}^{\tau }\;[dN_{ij}(t)-Y_{i}(t)\exp\{\beta^{\text{T}}Z_{ij}(t)\}\text{d}\mu_{0j}(t)]=0$$

one can obtain an empirical estimator ${\hat{\mu }}_{0j}^{r}(t;{\hat{\beta }}^{r},\tilde{\theta })$ for the baseline mean function $\mu_{0j}(t)$, where

(8) $${\hat{\mu }}_{0j}^{r}(t;{\hat{\beta }}^{r},\tilde{\theta })=n^{-1}\sum_{i=1}^{n}\int_{0}^{t}S_{j}^{\left(0\right)}{\left(t;{\hat{\beta }}^{r}\right)}^{-1}\;\text{d}N_{ij}^{r}(t;\tilde{\theta })$$

Note that, although *β* is denoted the same in the log-rate ratio model (4) and proportional rates model (1), the *β* in the model (4) may not be fully identifiable. The covariate with *β* in model (4) is $X_{ij}(t)=Z_{ij}(t)-Z_{iJ}(t)$, which is the difference between $Z_{ij}(t)$ and $Z_{iJ}(t)$. If a covariate, for example, age is included and has the common effect on the rate function for all event categories, then the corresponding *X_ij_* equals 0, and consequently, the corresponding component in *β* is not identifiable. Another situation is when a covariate is included in rate model for all event categories, but the effects are different for different event category. In this situation, what is estimable in *β* in model (4) is the difference between the effects of that covariate for different categories and not the effects themselves. Taking *J *=* *2 for example, one can write $Z_{i1}={\left({\tilde{Z}}_{i},0\right)}^{\text{T}}$ and $Z_{i2}={\left(0,{\tilde{Z}}_{i}\right)}^{\text{T}}$, where ${\tilde{Z}}_{i}$ is the covariate in the rates models for both event categories, for example, gender, and $\beta ={\left(\beta_{1},\beta_{2}\right)}^{\text{T}}$, where *β_j_* is the effect of ${\tilde{Z}}_{i}$ on rate function for event category *j* for *j *=* *1, 2. The difference between $Z_{i1}$ and $Z_{i2}$ is $X_{i1}(t)={\left({\tilde{Z}}_{i},-{\tilde{Z}}_{i}\right)}^{\text{T}}$, and one can write $\beta^{\text{T}}X_{i1}(t)=(\beta_{1}-\beta_{2}){\tilde{Z}}_{i}$. From this expression, we can see that only the contrast $\beta_{1}-\beta_{2}$ can be estimated from model (4), not *β*_1_ and *β*_2_ individually. Including some same set of covariates in the rate models for some event categories is common in practice. Therefore, it is not feasible to use the log-likelihood function (6) to estimate *β* in general. However, even though *β* in model (4) is not identifiable, our proposed method can still work because $p_{ij}(t)$ can still be consistently estimated using function (6).

Also note that the proportional rates model (1) with a baseline rate function specific for each event type is quite general. One may restrict the model with the baseline rate function to be proportional to a reference category *J*, meaning $d\mu_{0j}(t)=\gamma_{0j}d\mu_{0}(t)$, where $d\mu_{0}(t)$ is the baseline rate function of the reference category. The model can be written as

(9) $$E\{dN_{ij}^{*}(t)|Z_{ij}(t),{△}_{j}\}=\exp\{\beta_{0}^{\text{T}}Z_{ij}(t)+\gamma_{0}^{\text{T}}{△}_{j}\}\text{d}\mu_{0}(t)$$

where ${△}_{j}$ is a column vector of 1 in the *j*th element and 0 otherwise with $\gamma_{0}={\left(\gamma_{01},\ldots ,\gamma_{0(J-1)}\right)}^{\text{T}}$ as the corresponding coefficient. One can show that the formula (5) becomes

(10) $$\log\{p_{ij}^{c}(t)/p_{iJ}^{c}(t)\}=\beta_{0}^{\text{T}}X_{ij}(t)+\gamma_{0j}+\kappa_{j}(t|Z_{ij})$$

and estimate *β*_0_ and $\gamma_{0j}$ using completely observed data via parametric estimating equations

(11) $$\sum_{i=1}^{n}\sum_{j=1}^{J}\int_{0}^{\tau }X_{ij}(t)\{\delta_{ij}(t)-p_{ij}^{c}(t;\beta ,\gamma )\}R_{i}(t)\;\text{d}N_{i\cdot }(t)=0$$

where

$$p_{ij}^{c}(t;\beta ,\gamma )=\frac{\exp\{\beta^{\text{T}}X_{ij}(t)+\gamma_{0j}\}}{\sum_{\ell =1}^{J}\exp\{\beta^{\text{T}}X_{i\ell }(t)+\gamma_{0\ell }\}}$$

assuming $\kappa_{j}(t|Z_{ij})=0$ for j=1,…,J.

With ${\hat{\beta }}^{c}$ and ${\hat{\gamma }}^{c}$ solving equation (11), one can obtain a more efficient estimator for *β*_0_ and *γ*_0_ using all of the events by replacing the unknown quantity $\delta_{ij}(t)$ with $p_{ij}^{c}(t;{\hat{\beta }}^{c},{\hat{\gamma }}^{c})$. This yields the estimating equations

(12) $$U^{r}(\beta ,\gamma )=\sum_{i=1}^{n}\sum_{j=1}^{J}\int_{0}^{\tau }\{W_{ij}(t)-\bar{W}(t;\beta ,\gamma )\}\;\text{d}N_{ij}^{r}(t;{\hat{\beta }}^{c},{\hat{\gamma }}^{c})=0$$

where $W_{ij}(t)={\left(Z_{ij}^{\text{T}}(t),{△}_{j}^{\text{T}}\right)}^{\text{T}},\;\bar{W}(t;\beta ,\gamma )=S^{\left(1\right)}(t;\beta ,\gamma )/S^{\left(0\right)}(t;\beta ,\gamma )$ with

$$S^{\left(d\right)}(t;\beta ,\gamma )=n^{-1}\sum_{i=1}^{n}\sum_{j=1}^{J}Y_{i}(t)W_{ij}{\left(t\right)}^{⊗d}\exp\{\beta^{\text{T}}Z_{ij}(t)+\gamma^{\text{T}}{△}_{j}\}$$

for *d *=* *0, 1, and

$$dN_{ij}^{r}(t;\beta ,\gamma )=R_{i}(t)dN_{ij}(t)+\{1-R_{i}(t)\}p_{ij}(t;\beta ,\gamma )dN_{i\cdot }(t)$$

By comparing models (5) and (10), one can conduct a statistical test for the proportionality of the baseline rate functions by testing if $\eta_{0j}(t)$ is constant for all *t*, i.e., testing the null hypothesis $H_{0}:\eta_{0j}(t)=\gamma_{0j}$ for t≥0. Using our approach, the null hypothesis is equivalent to $H_{0}:\xi_{j1}=\ldots =\xi_{j(m+3)}=0$ for each *j*, which can be tested via a Wald-type test procedure. Under a more restricted model (9), the fully parametric model (10) for $p_{ij}(t)$ is somewhat different from the one proposed by Schaubel and Cai.^5^ The fully parametric model for $p_{ij}(t)$ in Schaubel and Cai^5^ includes more covariates than model (10), such as time of event occurrence *t* and number of previous events $N_{i\cdot }(t-)$.

## 3 Asymptotic theory

Large sample properties of the semiparametric estimators ${\hat{\beta }}^{r}$ and ${\hat{\mu }}_{0j}^{r}$ using model (5) will be derived in this section. The developments are more challenging than those in Schaubel and Cai,^5^ which covers only the more restrictive parametric model (10). We first state our notations. Let

$$p_{ij}^{c}(t;\theta )=\exp\{m_{ij}^{c}(t;\theta )\}/\sum_{\ell =1}^{J}\exp\{m_{i\ell }^{c}(t;\theta )\}$$

and let

$${\dot{p}}_{ij}^{c}(t;\theta )=\partial p_{ij}^{c}(t;\theta )/\partial \theta =p_{ij}^{c}(t;\theta )\{{\tilde{X}}_{ij}(t)-\sum_{\ell =1}^{J}{\tilde{X}}_{i\ell }(t)p_{i\ell }^{c}(t;\theta )\}$$

where ${\tilde{X}}_{ij}(t)={\left(X_{ij}{\left(t\right)}^{\text{T}},B_{j}{\left(t\right)}^{\text{T}}\right)}^{\text{T}}$. The score function of $n^{-1}\ell (\theta )$ can be written as $U(\theta )=n^{-1}\sum_{i=1}^{n}\sum_{j=1}^{J}U_{ij}(\theta )$, where

$$U_{ij}(\theta )=\int_{0}^{\tau }{\tilde{X}}_{ij}(t)\{\delta_{ij}(t)-p_{ij}^{c}(t;\theta )\}R_{i}(t)\;\text{d}N_{i\cdot }(t)$$

and the negative Hessian matrix of $n^{-1}\ell (\theta )$ can be written as

$$H(\theta )=n^{-1}\sum_{i=1}^{n}\sum_{j=1}^{J}\int_{0}^{\tau }{\tilde{X}}_{ij}(t){\dot{p}}_{ij}^{c}(t;\theta )R_{i}(t)\;\text{d}N_{i\cdot }(t)$$

Regularity conditions, especially for the number of interior knots, are outlined here. These conditions are required for the proof of the large sample properties of our estimators.

- Variables ${\left\{N_{ij}(\cdot ),Y_{ij}(\cdot ),Z_{ij}(\cdot )\right\}}_{j=1}^{J}$ (i=1,…,n) are independent and identically distributed.
- The distribution of censoring time *C_i_* satisfies $P(C_{i}\geq \tau )>0$ for each *i*.
- The sample path of the covariates satisfies $|Z_{ij\ell }(0)|+\int_{0}^{\tau }|dZ_{ij\ell }(t)|<c_{Z}<\infty$ for every ℓ, where $Z_{ij\ell }$ is the ℓth element of the covariate *Z_ij_*.
- The limiting matrices $\Omega (\beta_{0})$ and $H(\theta_{0})$ are positive-definite.
- The baseline rate functions $d\mu_{0j}(\cdot ),\;j=1,\ldots ,J$ are bounded away from zero and infinity on [0,τ].
- The second derivative of $\eta_{0j}(\cdot )$ exists and satisfies Lipschitz condition of order *ϵ* on [0,τ] for j=1,…,J for some ϵ∈(0,1].
- The number of interior knots satisfies $n^{1/(4+2\epsilon )}<m<n^{1/4}$.

Note that Conditions (a)–(e) are regularity conditions for recurrent event processes, outlined in Cai and Schaubel.^6^ The smoothness condition in (f) is similar to the condition (C1) in Wang et al.^11^ and enables estimation of $\eta_{0j}(t)$ using spline functions, with Condition (g) describing the number of parameters used in the spline functions relative to the sample size.

According to Wang et al.,^11^ the convergence rate of the estimator of *β*_0_ in model (5) is $n^{1/2}$ under Conditions (c)–(g), while the convergence rate of the nonparametric estimator of $\eta_{0j}(t)$ is slower than $n^{1/2}$. This makes that the convergence rate of the estimator for $p_{ij}(t)$ is slower than $n^{1/2}$. However, we can show that the convergence rate of our estimator for the regression parameter *β*_0_ is $n^{1/2}$. The following theorem describes the large sample theory of our estimator. The detailed proof is given in Appendix 1.

Theorem 1 *Under Conditions (a)–(g), the estimator*${\hat{\beta }}^{r}$*is a consistent estimator of β_0_ and*$n^{1/2}({\hat{\beta }}^{r}-\beta_{0})$*converges in distribution to a normal variable with mean 0 and variance Σ, which can be consistently estimated by*$\hat{\Omega }{\left({\hat{\beta }}^{r}\right)}^{-1}\hat{\Phi }({\hat{\beta }}^{r})\hat{\Omega }{\left({\hat{\beta }}^{r}\right)}^{-1}$*, where*

$$\hat{\Omega }(\beta )=n^{-1}\sum_{i=1}^{n}\sum_{j=1}^{J}\int_{0}^{\tau }\left\{S_{j}^{\left(2\right)}(t;\beta )/S_{j}^{\left(0\right)}(t;\beta )-{\bar{Z}}_{j}{\left(t;\beta \right)}^{⊗2}\right\}\;\text{d}N_{ij}^{r}(t;\tilde{\theta })$$

and

$$\hat{\Phi }(\beta )=n^{-1}\sum_{i=1}^{n}{\hat{\Psi }}_{i}{\left(\beta ,\tilde{\theta }\right)}^{⊗2}$$

with

$$\begin{array}{l} {\hat{\Psi }}_{i}(\beta ,\theta )=\sum_{j=1}^{J}\int_{0}^{\tau }\{Z_{ij}(t)-{\bar{Z}}_{j}(t;\beta )\}d{\hat{M}}_{ij}^{r}(t;\beta ,\theta )+\hat{\Gamma }(\beta ,\theta )H{\left(\theta \right)}^{-1}U_{ij}(\theta ),\\ d{\hat{M}}_{ij}^{r}(t;\beta ,\theta )=dN_{ij}^{r}(t;\theta )-Y_{i}(t)\exp\{\beta^{\text{T}}Z_{ij}(t)\}d{\hat{\mu }}_{0j}^{r}(t;\beta ,\theta ), \end{array}$$

and

$$\hat{\Gamma }(\beta ,\theta )=n^{-1}\sum_{i=1}^{n}\sum_{j=1}^{J}\int_{0}^{\tau }\{Z_{ij}(t)-{\bar{Z}}_{j}(t;\beta )\}\rho_{ij}(t;\theta ){\tilde{X}}_{ij}{\left(t\right)}^{\text{T}}\{1-R_{i}(t)\}dN_{i\cdot }(t)$$

where $\rho_{ij}(t;\theta )=p_{ij}(t;\theta )\{1-p_{ij}(t;\theta )\}$.

The consistency of ${\hat{\beta }}^{r}$ can be proved via consistency of $\tilde{\theta }$ and conventional convex theories. Large sample normality can be established via an approximation to $n^{1/2}({\hat{\beta }}^{r}-\beta_{0})$ by a summation of independent and identical random vectors, as shown in Appendix 1. Note that the variation of ${\hat{\beta }}^{r}$ is larger than that for ${\hat{\beta }}^{n}$ which solves equation (2) assuming that there are no missing data, since the estimation for $p_{ij}(t)$ creates additional uncertainty when the event type is missing. This additional variation can be seen in the second term of ${\hat{\Psi }}_{i}(\beta ,\theta )$. Empirical studies show that the efficiency loss compared to the estimator with no missing data may be rather small.

Let $A_{j}(t;\beta ,\theta )=-\int_{0}^{t}{\bar{Z}}_{j}(s;\beta )d{\hat{\mu }}_{0j}(s;\beta ,\theta ),$ and

$$D_{j}(t;\beta ,\theta )=n^{-1}\sum_{i=1}^{n}\int_{0}^{t}S_{j}^{\left(0\right)}{\left(s;\beta \right)}^{-1}\rho_{ij}(s;\theta ){\tilde{X}}_{ij}(s)\{1-R_{i}(s)\}\;\text{d}N_{i\cdot }(s)$$

The following theorem describes the limiting properties of the baseline mean function estimator ${\hat{\mu }}_{0j}^{r}(t;{\hat{\beta }}^{r},\tilde{\theta })$ for $\mu_{0j}(t)$ in model (1).

Theorem 2 *Under the same conditions of Theorem 1, the baseline mean function estimator* ${\hat{\mu }}_{0j}^{r}(t;{\hat{\beta }}^{r},\tilde{\theta })$ *is uniformly consistent for* $\mu_{0j}(t),\;t\in [0,\tau ]$ *, and* $n^{1/2}\{{\hat{\mu }}_{0j}^{r}(t;{\hat{\beta }}^{r},\tilde{\theta })-\mu_{0j}(t)\}$ *converges weakly to a Gaussian process with mean 0 and covariance function* $V_{j}(s,t),\;s,t\in [0,\tau ]$ *, which can be consistently estimated by*

(13) $${\hat{V}}_{j}^{r}(s,t)=n^{-1}\sum_{i=1}^{n}{\hat{\phi }}_{ij}(s;{\hat{\beta }}^{r},\tilde{\theta }){\hat{\phi }}_{ij}(t;{\hat{\beta }}^{r},\tilde{\theta })$$

where

$$\begin{array}{l} {\hat{\phi }}_{ij}(t;\beta ,\theta )=A_{j}{\left(t;\beta ,\theta \right)}^{\text{T}}\hat{\Omega }{\left(\beta \right)}^{-1}{\hat{\Psi }}_{i}(\beta ,\theta )\\ \;+D_{j}(t;\beta ,\theta )H{\left(\theta \right)}^{-1}\sum_{j=1}^{J}U_{ij}(\theta )+\int_{0}^{t}S_{j}^{\left(0\right)}{\left(s;\beta \right)}^{-1}\;\text{d}{\hat{M}}_{ij}^{r}(s;\beta ,\theta ) \end{array}$$

The proof begins by decomposing ${\hat{\omega }}_{j}(t)={\hat{\mu }}_{0j}^{r}(t;{\hat{\beta }}^{r},\tilde{\theta })-\mu_{0j}(t)$ as ${\hat{\omega }}_{j}^{\left(1\right)}(t)+{\hat{\omega }}_{j}^{\left(2\right)}(t)$, where ${\hat{\omega }}_{j}^{\left(1\right)}(t)={\hat{\mu }}_{0j}^{r}(t;{\hat{\beta }}^{r},\tilde{\theta })-{\hat{\mu }}_{0j}^{r}(t;\beta_{0},\theta_{0})$ and ${\hat{\omega }}_{j}^{\left(2\right)}(t)={\hat{\mu }}_{0j}^{r}(t;\beta_{0},\theta_{0})-\mu_{0j}(t)$. The uniform consistency of ${\hat{\mu }}_{0j}^{r}(t;{\hat{\beta }}^{r},\tilde{\theta })$ can be proved by showing that both $\underset{t\in [0,\tau ]}{\sup}|{\hat{\omega }}_{j}^{\left(1\right)}(t)|$ and $\underset{t\in [0,\tau ]}{\sup}|{\hat{\omega }}_{j}^{\left(2\right)}(t)|$ converge to 0. The uniform convergence of ${\hat{\omega }}_{j}^{\left(2\right)}(t)$ can be proved using a law of large numbers for empirical processes and uniform convergence of ${\tilde{\eta }}_{0j}$ to $\eta_{0j}$. The uniform convergence of ${\hat{\omega }}_{j}^{\left(1\right)}(t)$ involves some additional assumptions. The details of the proof are provided in Appendix 1. The proof of weak convergence, which establishes tightness and convergence to finite-dimensional distributions, follows the standard tools in Pollard^14^ and van der Vaart and Wellner;^15^ see Appendix 1 for details.

## 4 Simulation study

In this section, we demonstrate the feasibility of our proposed method via comprehensive simulations. We first examine a scenario when the proportional baseline rate model (9) holds; therefore, the general model (1) also holds. We then investigate a scenario when the baseline rate functions are nonproportional, in that model (1) holds, but not model (9). For subject *i*, two types of recurrent events were simulated from two intensity functions sharing the same latent variable *G_i_*, which was sampled from Gamma(1/α,α) with $E(G_{i})=1$ and var$(G_{i})=\alpha$. In the first scenario, the intensity functions are assumed $\lambda_{i1}(t)=G_{i}r_{01}t\exp(\beta_{1}{\tilde{Z}}_{i})$ and $\lambda_{i2}(t)=G_{i}r_{02}t\exp(\beta_{2}{\tilde{Z}}_{i})$ with constants *r*_01_ and *r*_02_, while, in the second scenario, the intensity functions are $\lambda_{i1}(t)=G_{i}r_{01}\exp(\beta_{1}{\tilde{Z}}_{i})$ and $\lambda_{i2}(t)=G_{i}r_{02}h_{0}(t)\exp(\beta_{2}{\tilde{Z}}_{i})$, where $h_{0}(t)=\exp\{-\sin(t/3)-3\cos(3t)\}$. We let *α* = 0, 0.5, or 1 for different dependencies between two types of recurrent events, where *α* = 0 indicates two types of recurrent events are independent. We set $r_{01}=0.125$ and $r_{02}=0.0625$ in the first scenario and $r_{01}=r_{02}=0.125$ in the second scenario. We let $\beta_{1}=0$ or log⁡(2) and $\beta_{2}=0$. The covariate ${\tilde{Z}}_{i}$ was randomly drawn from a Bernoulli distribution with probability 0.5. The censoring time was generated uniformly between 0 and 5 for each subject. In summary, there are 1.2–1.4 total number of events on average in these simulation scenarios, with a 1:2 or 1:3 ratio of type 1 events to type 2 events. The maximum number of events in a subject ranges from 6 to 11 events on average among the scenarios.

One can show that the rate functions can be expressed as $E\{dN_{ij}^{*}(t)|Z_{ij}(t)\}=\exp(\beta_{0}^{\text{T}}Z_{ij})d\mu_{0j}(t)$, where $\beta_{0}={\left(\beta_{1},\beta_{2}\right)}^{\text{T}},\;Z_{ij}={\left(I(j=1){\tilde{Z}}_{i},I(j=2){\tilde{Z}}_{i}\right)}^{\text{T}},\;d\mu_{01}(t)=r_{01}t$ and $d\mu_{02}(t)=r_{02}t$ in the first scenario, and $d\mu_{01}(t)=r_{01}$ and $d\mu_{02}(t)=r_{02}h_{0}(t)$ in the second scenario. Note that the weighted estimating equations method in Schaubel and Cai^5^ is unbiased in the first scenario if one uses ${\tilde{Z}}_{i}$ as the covariate in the logistic regression model for $p_{i1}(t)$. However, it is quite evident that the model is misspecified if one uses the same model in the second scenario.

We assumed that the probability of having a missing category was given by

$$1-\pi_{i}(t)={\left[1+\exp\{-\epsilon^{\prime }z_{i}(t)\}\right]}^{-1}$$

where $z_{i}(t)=(1,t,N_{i\cdot }(t-),{\tilde{Z}}_{i})\prime$. We let $\epsilon =(\epsilon_{0},\epsilon_{t},\epsilon_{n},\epsilon_{z})\prime$, with $\epsilon_{t}=-0.15,\;\epsilon_{n}=0.1$, and $\epsilon_{z}=0,\log(2)$, where $\epsilon_{z}=0$ indicated that the missingness depends on covariates and the missingness assumption is MAR. Various values of *ϵ*_0_ were given to control the percentages of events with missing categories, denoted by $M_{p}$. Here, we assume that the missingness does not depend on the event type, i.e., $\pi_{i1}=\pi_{i2}=\pi_{i}$ and $\kappa_{1}=0$.

Table 1 shows the simulation results for *β*_1_ under 1,000 repetitions of sample size *n *=* *200. We present the results of our proposed estimator ${\hat{\beta }}_{1}^{r}$ and the weighted estimating equations method ${\hat{\beta }}_{1}^{w}$, where $z_{i}(t)$ was used as a covariate in the logistic regression model for $p_{i1}(t)$ to derive ${\hat{\beta }}_{1}^{w}$. We also present the results of the estimator ${\hat{\beta }}_{1}^{n}$ assuming that there is no missing event category. This approach is generally not feasible in practice but provides the best possible results in the ideal situation. Note that our proposed estimator is obtained under a more general model (1). Later, we will show that our estimator endures little efficiency loss even when the underlying model has proportional baseline rates. We report the average of biases in our replicated estimates, empirical standard deviation *σ*_1_, and the relative mean-squared error to our proposed method, denoted by $e_{r}^{x}=m^{x}/m^{r}$, where $m^{x}={\left({\hat{\beta }}_{1}^{x}-\beta_{1}\right)}^{2}+{\left(\sigma_{1}^{x}\right)}^{2},\;x=n,r,w$. The result shows that our estimator provides comparable estimates when the weighted estimating equations method correctly specifies the model in the first scenario. The efficiency loss is minimal, as the relative mean-squared errors are all close to 1. When the model is misspecified by the weighted estimating equations method in the second scenario, the estimator has a relatively larger variation, especially when more events with missing type are present in the data. Our estimator on the other hand provides a more robust approach with significant efficiency improvements. Overall, our proposed estimator does not lose much efficiency compared to the ideal solution, while outperforming the current existing parametric estimator.

**Table 1.** Simulation results are reported based on scenario 1, where data were generated from model (9), and scenario 2, where data were generated from model (1).

					${\hat{\beta }}_{1}-\beta_{1}$			***σ*_1_**			Ratio	
Scenario	*β*_1_	*α*	*ϵ_z_*	$M_{p}$ (%)	${\hat{\beta }}_{1}^{n}$	${\hat{\beta }}_{1}^{r}$	${\hat{\beta }}_{1}^{w}$	${\hat{\beta }}_{1}^{n}$	${\hat{\beta }}_{1}^{r}$	${\hat{\beta }}_{1}^{w}$	$e_{r}^{n}$	$e_{r}^{w}$
1	0.69	0.5	0	10	0.008	0.009	0.010	0.230	0.233	0.233	0.97	1.00
				20		0.011	0.011		0.237	0.236	0.95	0.99
				30		0.010	0.011		0.242	0.241	0.90	0.99
			0.69	10		0.010	0.010		0.234	0.234	0.97	1.00
				20		0.011	0.011		0.238	0.237	0.93	0.99
				30		0.011	0.011		0.241	0.240	0.91	0.99
		1.0	0	10	0.010	0.011	0.011	0.257	0.260	0.260	0.98	1.00
				20		0.012	0.012		0.260	0.260	0.98	1.00
				30		0.012	0.012		0.263	0.262	0.96	0.99
			0.69	10		0.012	0.012		0.260	0.259	0.98	1.00
				20		0.012	0.012		0.261	0.260	0.97	0.99
				30		0.013	0.013		0.261	0.260	0.97	0.99
2	0	0	0	20	–0.001	0.001	–0.003	0.249	0.267	0.279	0.87	1.09
				30		0.003	–0.007		0.280	0.300	0.80	1.15
				40		0.006	–0.005		0.294	0.322	0.72	1.20
			0.69	20		0.001	–0.005		0.265	0.280	0.88	1.11
				30		0.005	–0.005		0.278	0.297	0.81	1.14
				40		–0.006	–0.008		0.297	0.323	0.71	1.19
		0.5	0	20	–0.010	–0.015	–0.018	0.287	0.307	0.314	0.88	1.05
				30		–0.013	–0.020		0.321	0.339	0.80	1.12
				40		–0.013	–0.027		0.325	0.346	0.78	1.14
			0.69	20		–0.017	–0.021		0.307	0.316	0.87	1.06
				30		–0.015	–0.024		0.315	0.330	0.83	1.10
				40		–0.016	–0.022		0.333	0.357	0.75	1.15
3	0.69	0	0	20	0.002	0.008	0.007	0.222	0.238	0.245	0.87	1.06
				30		0.006	0.007		0.251	0.263	0.78	1.10
				40		0.005	0.006		0.266	0.281	0.70	1.12
			0.69	20		0.005	0.005		0.234	0.244	0.90	1.08
				30		0.007	0.008		0.247	0.258	0.81	1.09
				40		0.002	0.007		0.257	0.270	0.75	1.11
		0.5	0	20	–0.001	–0.003	–0.003	0.248	0.264	0.273	0.89	1.07
				30		–0.004	0.000		0.270	0.287	0.85	1.13
				40		–0.004	–0.001		0.283	0.302	0.77	1.14
			0.69	20		–0.003	–0.004		0.259	0.269	0.92	1.07
				30		–0.002	0.000		0.270	0.282	0.85	1.10
				40		–0.004	–0.004		0.274	0.287	0.82	1.10

Table 2 demonstrates that the distribution of our estimator can be well approximated by a normal distribution in finite samples. We show the results based on model (1) in the second scenario when *n *=* *200 and 400. The results based on model (9) in the first scenario are similar; hence omitted here. As one can see, the average of our standard error estimates, denoted by ${\bar{\sigma }}_{1}$ and ${\bar{\sigma }}_{2}$, is close to the empirical standard deviation, *σ*_1_ and *σ*_2_, respectively, and the coverage rate $C_{p}$ for *β*_1_ based on the 95% confidence interval is close to the nominal level. Meanwhile, the size of the Wald-type test $C_{0}$ for $\beta_{2}=0$ is close to the given significance level at 0.05.

**Table 2.** Simulation results of parameter estimations from our proposed method for model (1).

					${\hat{\beta }}_{1}^{r}$				${\hat{\beta }}_{2}^{r}$			
$\left(\beta_{1},\beta_{2}\right)$	*α*	*ϵ_z_*	$M_{p}$ (%)	*n*	Bias	*σ*_1_	${\bar{\sigma }}_{1}$	$C_{p}$	Bias	*σ*_2_	${\bar{\sigma }}_{2}$	$C_{0}$
(0,0)	0	0	20	200	0.001	0.267	0.272	0.961	–0.006	0.149	0.151	0.035
				400	–0.002	0.185	0.191	0.959	–0.005	0.105	0.106	0.041
			30	200	0.003	0.280	0.287	0.961	–0.007	0.152	0.154	0.040
				400	–0.002	0.190	0.199	0.960	–0.005	0.107	0.108	0.045
			40	200	0.006	0.294	0.294	0.957	–0.007	0.153	0.155	0.038
				400	0.003	0.198	0.207	0.961	–0.006	0.109	0.109	0.049
		0.69	20	200	0.001	0.265	0.272	0.967	–0.006	0.149	0.151	0.041
				400	–0.003	0.184	0.191	0.964	–0.004	0.106	0.106	0.048
			30	200	0.005	0.278	0.282	0.961	–0.008	0.150	0.153	0.037
				400	–0.001	0.190	0.198	0.955	–0.005	0.107	0.108	0.046
			40	200	–0.006	0.297	0.295	0.950	–0.005	0.153	0.155	0.041
				400	–0.006	0.200	0.207	0.958	–0.004	0.109	0.110	0.050
	0.5	0	20	200	–0.015	0.307	0.297	0.948	0.000	0.195	0.188	0.067
				400	–0.004	0.212	0.207	0.944	–0.004	0.134	0.133	0.060
			30	200	–0.013	0.321	0.306	0.947	0.000	0.196	0.190	0.062
				400	–0.004	0.222	0.215	0.941	–0.004	0.135	0.134	0.057
			40	200	–0.013	0.325	0.317	0.951	–0.001	0.199	0.192	0.056
				400	–0.005	0.228	0.222	0.950	–0.004	0.136	0.136	0.057
		0.69	20	200	–0.017	0.307	0.297	0.946	0.000	0.194	0.188	0.062
				400	–0.005	0.213	0.207	0.948	–0.004	0.133	0.133	0.056
			30	200	–0.015	0.315	0.306	0.952	–0.001	0.197	0.190	0.064
				400	–0.003	0.220	0.214	0.945	–0.005	0.135	0.134	0.056
			40	200	–0.016	0.333	0.318	0.950	–0.001	0.198	0.192	0.064
				400	–0.010	0.234	0.223	0.950	–0.003	0.138	0.136	0.059
(0.69,0)	0	0	20	200	0.008	0.238	0.234	0.958	–0.006	0.158	0.152	0.060
				400	0.002	0.163	0.164	0.948	–0.001	0.110	0.107	0.063
			30	200	0.006	0.251	0.246	0.957	–0.005	0.164	0.157	0.065
				400	0.003	0.170	0.170	0.952	–0.001	0.111	0.109	0.053
			40	200	0.005	0.266	0.251	0.947	–0.004	0.167	0.158	0.068
				400	0.004	0.177	0.177	0.950	–0.002	0.113	0.111	0.059
		0.69	20	200	0.005	0.234	0.232	0.955	–0.006	0.159	0.152	0.066
				400	0.000	0.160	0.163	0.954	–0.001	0.110	0.107	0.061
			30	200	0.007	0.247	0.239	0.948	–0.007	0.163	0.155	0.064
				400	0.002	0.168	0.168	0.950	–0.002	0.112	0.109	0.056
			40	200	0.002	0.257	0.248	0.954	–0.005	0.169	0.159	0.067
				400	0.002	0.173	0.174	0.956	–0.001	0.115	0.112	0.060
	0.5	0	20	200	–0.003	0.264	0.260	0.952	0.004	0.201	0.189	0.071
				400	–0.004	0.178	0.183	0.956	0.001	0.136	0.133	0.058
			30	200	–0.004	0.270	0.268	0.950	0.004	0.203	0.191	0.068
				400	–0.006	0.183	0.189	0.961	0.001	0.138	0.135	0.055
			40	200	–0.004	0.283	0.277	0.946	0.004	0.204	0.193	0.064
				400	–0.005	0.191	0.195	0.960	0.001	0.140	0.137	0.049
		0.69	20	200	–0.003	0.259	0.259	0.952	0.004	0.201	0.189	0.070
				400	–0.005	0.176	0.182	0.964	0.001	0.137	0.134	0.060
			30	200	–0.002	0.270	0.265	0.955	0.003	0.201	0.191	0.068
				400	–0.005	0.180	0.187	0.962	0.000	0.138	0.135	0.066
			40	200	–0.004	0.274	0.272	0.952	0.003	0.205	0.193	0.063
				400	–0.004	0.184	0.191	0.962	–0.001	0.140	0.137	0.055

As seen in Tables 1 and 2, our estimator is robust to the MAR assumption when $\epsilon_{z}\neq 0$. Both point and variance estimation are consistent. In fact, the efficiency is slightly better compared to the estimator when $\epsilon_{z}=0$. We also set different values of *κ*_1_ to examine the performance of our estimation method under the missingness not at random assumption. However, since the simulation results are similar, we do not report the results here.

## 5 CF registry data

CF is one of the most common life-shortening, autosomal recessive genetic disorders, affecting about 30,000 individuals in the United States.^16^ It is caused by mutations in the gene encoding the CF transmembrane conductance regulator.^17^ Chronic lung infection and associated inflammation lead to significant morbidity in CF, with respiratory failure the leading cause of mortality. PA, one of the major virulent pathogens in CF patients, is a well-known risk factor for CF lung disease progression and survival. Several baseline risk factors for PA acquisition were examined in Lai et al.^18^ Meconium ileus, late CF diagnosis through signs and symptoms, severe CF genotypes, and female gender are associated with a higher risk of acquiring PA. However, most of the PA cases examined in the study were initial infections, which may be transient and less predictive of negative outcomes. On the other hand, mucoid PA, which is thought to develop after recurrent infections, is likely more critical to a patient’s lung disease progression.^19^ Therefore, regression modeling for different PA types, i.e., mucoid and nonmucoid, is important, since the baseline risk factors may differentially impact different infection types in various manners.

In this section, we extended the analysis in Lai et al.^18^ to multiple event types using the United States 2016 CF Foundation Patient Registry (CFFPR), in which baseline characteristics, such as genotype, phenotype, and other prognosis factors, are recorded upon enrollment. The CFFPR documents the diagnosis and follow-up of 29,887 individuals with CF in the registry. We aim to model the nonmucoid and mucoid PA occurrence rates in association with three baseline risk factors, which include (1) gender, (2) genotype, categorized based on the most common mutation: F508del homozygous, F508del heterozygous, and neither or unknown, and (3) method of diagnosis, categorized in four groups described elsewhere:^18^ newborn screening, meconium ileus, family history without symptom, and symptom and sign. Medication use for chronic PA infections and study site/center is included in the model for adjustment of possible confounding effects.

In the 2016 registry, we identified 14,888 patients who were born after 1997 and had complete baseline risk data in 188 accredited CF centers. In summary, half of these patients were male, 47% were F508del homozygous, 39% were F508del heterozygous, 47% were diagnosed by newborn screening, 31% were diagnosed by emerging symptoms and signs, and 19% and 3% were diagnosed by meconium ileus and family history, respectively. In the follow-up visits, there were 27,288 nonmucoid and 6,323 mucoid PA infections, in addition to 5,445 culture positives for both nonmucoid and mucoid types at the same visit. Since our method assumes two kinds of events cannot occur simultaneously, we treated those visits with both infections as the third type of recurrent event. Meanwhile, there were 5,392 culture positives in PA but with unknown status, which is in a high frequency of missing event type (12% of the total events). Addressing the missing information is highly desirable.

Table 3 shows the estimation results by the complete-case analysis and our proposed rate proportion method, assuming that the missingness does not depend on the event category, i.e., $\kappa_{1}=\kappa_{2}=0$. We used AIC to choose the number of interior knots in the B-spline function in model (5). Up to five interior knots were examined, the minimum value of AIC was achieved by using a cubic function without any interior knots. We report rate ratio, exp⁡(β), and its 95% confidence interval (95% CI) with Wald-type test *p*-value with respect to a reference group. We also report the *p*-value, indicated with a dagger, for the overall comparison among levels in genotype and diagnostic method.

**Table 3.** Summary table for the complete-case analysis and rate proportion method.

	Complete-case			Rate proportion		
Covariates	exp⁡(β)	95% CI	*p*-value	exp⁡(β)	95% CI	*p*-value
	Nonmucoid PA infection					
Female	1.09	1.03, 1.15	0.001	1.08	1.01, 1.15	0.017
Genotype F508del			<0.001^a^			<0.001^a^
Homozygous	1.00	–	–	1.00	–	–
Heterozygous	0.89	0.83, 0.94	<0.001	0.89	0.84, 0.94	<0.001
Neither or unknown	0.89	0.77, 1.03	0.121	0.90	0.83, 0.97	0.008
Diagnostic method			0.079^a^			0.025†
Newborn screening	1.00	–	–	1.00	–	–
Meconium ileus	1.15	0.99, 1.34	0.069	1.10	1.01, 1.21	0.037
Family history	0.99	0.77, 1.27	0.946	0.94	0.80, 1.10	0.445
Symptom	1.13	0.93, 1.35	0.212	1.07	0.98, 1.16	0.127
Medication	1.85	1.64, 2.09	<0.001	1.88	1.68, 2.09	<0.001
	Mucoid PA infection					
Female	1.18	1.01, 1.38	0.038	1.16	1.08, 1.24	<0.001
Genotype F508del			0.298^a^			0.089†
Homozygous	1.00	–	–	1.00	–	–
Heterozygous	0.95	0.81, 1.12	0.547	0.95	0.86, 1.06	0.374
Neither or unknown	1.20	0.91, 1.59	0.199	1.21	1.00, 1.46	0.052
Diagnostic method			0.004^a^			<0.001†
Newborn screening	1.00	–	–	1.00	–	–
Meconium ileus	1.18	0.91, 1.54	0.212	1.13	0.99, 1.29	0.063
Family history	1.45	0.96, 2.21	0.079	1.38	1.02, 1.87	0.035
Symptom	1.59	1.17, 2.15	0.003	1.51	1.30, 1.75	<0.001
Medication	3.06	2.57, 3.66	<0.001	2.98	2.71, 3.28	<0.001
	Both PA infection					
Female	1.22	1.03, 1.44	0.018	1.21	1.12, 1.31	<0.001
Genotype F508del			0.287^a^			0.217†
Homozygous	1.00	–	–	1.00	–	–
Heterozygous	0.92	0.78, 1.09	0.341	0.93	0.81, 1.06	0.258
Neither or unknown	1.11	0.87, 1.42	0.388	1.13	0.90, 1.42	0.294
Diagnostic method			<0.001^a^			<0.001^a^
Newborn screening	1.00	–	–	1.00	–	–
Meconium ileus	1.62	1.24, 2.11	<0.001	1.54	1.32, 1.80	<0.001
Family history	1.91	1.11, 3.28	0.019	1.78	1.23, 2.58	0.002
Symptom	1.99	1.47, 2.70	<0.001	1.88	1.68, 2.11	<0.001
Medication	2.47	1.98, 3.08	<0.001	2.39	2.13, 2.68	<0.001

a Refer to the overall comparison among levels in genotype and diagnostic method.

As one can see, in the nonmucoid PA acquisition, both methods agree that female gender and heterozygous F508del genotype are significantly associated with the infection rate. While female has around 8% higher infection rate than male, patients with heterozygous F508del genotype have 11% lower infection rate than those with homozygous F508del genotype. However, the two methods have different test results in neither F508del nor unknown genotype and diagnosed by meconium ileus. While both methods have similar rate ratios, our proposed method, with more data points involved, has a narrower confidence interval and significant test result, which leads one to conclude that patients with a mild genotype (heterozygous F508del, neither F508del genotype, or unknown) have 10% lower nonmucoid PA infection rate than those with a more severe genotype (homozygous F508del), and patients diagnosed by with meconium ileus have 10% higher infection rate than those diagnosed by newborn screening. The finding is consistent with Lai et al.^18^ and other scientific reports. The disparity between the complete-case analysis and our method can be considered as an evidence against the missingness completely at random assumption. In fact, based on a multiple logistic regression model for the likelihood of missingness, the probability of missing event type is significantly associated with age, gender, genotype, and frequency of previous events. The results demonstrate that missing event type is more likely to occur in younger patients, in female patients, in patients with mild genotypes, and in patients with more prior PA infections.

Two methods otherwise have similar results in the mucoid PA acquisition and in both the types of infection in the same culture. One difference is that patients diagnosed by family history without symptoms are statistically significant in the mucoid PA infection using our proposed method, but not significant using the complete-case analysis.

**Figure 1.**
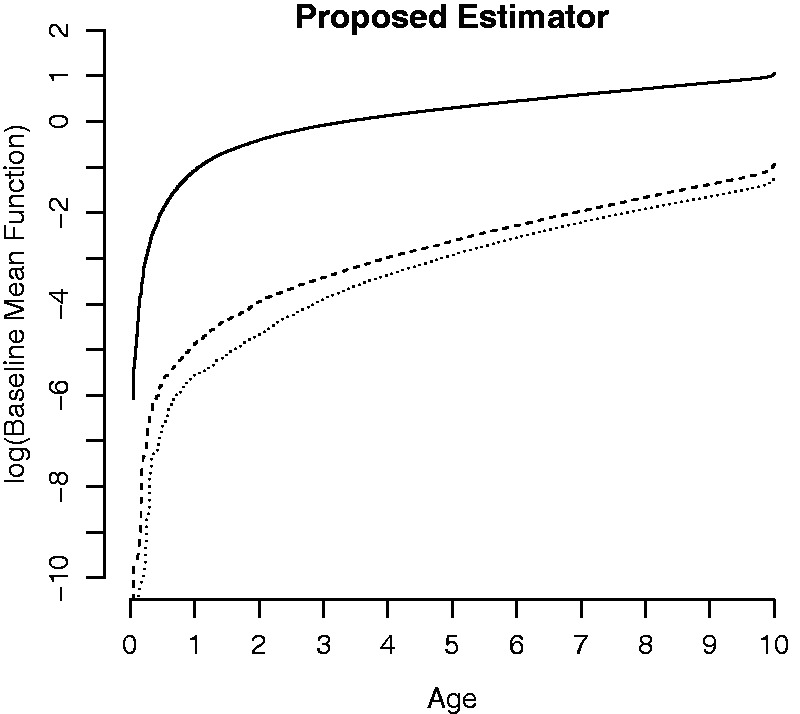
The baseline mean function estimation by our proposed method is shown in log-scale for nonmucoid (solid), mucoid (dash), and both (dot) PA infections.

The baseline mean function estimation for the three types of infections by our proposed estimator is shown in Figure 1. The testing for the proportionality of the baseline rate functions based on the Wald-type test is significant with *p*-value <0.0001, suggesting that model (9) is not a good fit to our data. Hence, we only report the results for the model (1). We also assess the assumption of equal probability of missingness by assuming that the log-ratio of the missingness probability $\kappa_{j}(t|Z_{ij})$ in model (5) is possibly non-zero. Here, we implemented different values of *κ*_1_ and *κ*_2_, ranging from –1.5 to 1.5, to explore the sensitivity of the parameter estimation when the missingness is not at random. The result in the supplementary material shows that our estimation is quite robust to the violation of the MAR assumption, as the changes in the point and variance estimates of the regression coefficients are minimal, even when *κ*_1_ and *κ*_2_ are large.

## 6 Conclusion and discussion

It is worth noting that the same proportionality property exploited by our method was also discussed in an intensity-based recurrent event model^20^ and in competing risk models with missing or uncertain cause of failure.^21–24^ A semiparametric framework for the estimation of $p_{ij}(t)$ otherwise has never been explored. It is widely anticipated that the semiparametric estimation will be more robust if the underlying unknown function is indeed time-dependent, which is quite likely in practice with time to event data.

The estimation procedure in this paper is tailored for the proportional rates model. It may not be feasible for a nonproportional rates model since the ratio of the rate functions may not be log-linearly correlated with covariates. It would be of interest to develop a more general approach for different types of rate models. One possibility is to derive the probability of missingness and then inversely weigh the estimating equations for unbiased estimation. Along these lines, one may also utilize the nonparametric estimation for the rate function to construct a doubly robust estimator, providing additional protection against the model misspecification.

## Supplemental Material

sj-pdf-1-smm-10.1177_09622802211023975 - Supplemental material for Semiparametric estimation of the proportional rates model for recurrent events data with missing event category Click here for additional data file. Supplemental material, sj-pdf-1-smm-10.1177_09622802211023975 for Semiparametric estimation of the proportional rates model for recurrent events data with missing event category by Feng-Chang Lin, Jianwen Cai, Jason P Fine, Elisabeth P Dellon and Charles R Esther in Statistical Methods in Medical Research

## Appendix 1

### A.1. Proof of theorem 1

By Taylor expansion of $U^{r}(\beta )$ around *β*_0_, one can have $n^{1/2}({\hat{\beta }}^{r}-\beta_{0})=\hat{\Omega }{\left(\bar{\beta }\right)}^{-1}n^{-1/2}U^{r}(\beta_{0})$, where $\hat{\Omega }(\bar{\beta })=-n^{-1}\partial U^{r}(\beta )/\partial \beta {|}_{\beta =\bar{\beta }}$ with $\bar{\beta }$ lying between ${\hat{\beta }}^{r}$ and *β*_0_. Under Conditions (a)–(e), law of large numbers, consistency of ${\hat{\beta }}^{r}$ and $\tilde{\theta }$, and uniform convergence of ${\tilde{\eta }}_{0j}$, one can show that $\hat{\Omega }(\bar{\beta })$ converges to

$$\Omega (\beta_{0})=\sum_{j=1}^{J}\int_{0}^{\tau }v_{j}(t;\beta_{0})s_{j}^{\left(0\right)}(t;\beta_{0})\;\text{d}\mu_{0j}(t)$$

where for *d *=* *0, 1, 2,

$$v_{j}(t;\beta )=s_{j}^{\left(2\right)}(t;\beta )/s_{j}^{\left(0\right)}(t;\beta )-{\bar{z}}_{j}{\left(t;\beta \right)}^{⊗2}$$

$$s_{j}^{\left(d\right)}(t;\beta )=\underset{n\to \infty }{\lim}n^{-1}\sum_{i=1}^{n}E[Y_{i}(t)Z_{ij}{\left(t\right)}^{⊗d}\exp\{\beta^{\text{T}}Z_{ij}(t)\}]$$

and ${\bar{z}}_{j}(t;\beta )=s_{j}^{\left(1\right)}(t;\beta )/s_{j}^{\left(0\right)}(t;\beta ).$ Let $U^{r}(\beta_{0})=U_{1}^{r}(\beta_{0})+U_{2}^{r}(\beta_{0})$, where

$$U_{1}^{r}(\beta_{0})=\sum_{i=1}^{n}\sum_{j=1}^{J}\int_{0}^{\tau }\{Z_{ij}(t)-{\bar{Z}}_{j}(t;\beta_{0})\}\;\text{d}M_{ij}^{r}(t;\beta_{0})$$

with

$$dM_{ij}^{r}(t;\beta )=R_{i}(t)dN_{ij}(t)+\{1-R_{i}(t)\}p_{ij}(t)dN_{i\cdot }(t)-Y_{i}(t)\exp\{\beta^{\text{T}}Z_{ij}(t)\}d\mu_{0j}(t)$$

and

$$U_{2}^{r}(\beta_{0})=\sum_{i=1}^{n}\sum_{j=1}^{J}\int_{0}^{\tau }\{Z_{ij}(t)-{\bar{Z}}_{j}(t;\beta_{0})\}\{p_{ij}(t;\tilde{\theta })-p_{ij}(t)\}\{1-R_{i}(t)\}\;\text{d}N_{i\cdot }(t)$$

Again, using Taylor expansion, one can get $p_{ij}(t;\tilde{\theta })-p_{ij}(t)=\rho_{ij}(t;\bar{\theta })\{m_{ij}(t;\tilde{\theta })-m_{ij}(t)\},$ where $m_{ij}(t)=\beta_{0}^{\text{T}}X_{ij}(t)+\eta_{0j}(t)$ and $\bar{\theta }=(\bar{\beta },\bar{\xi })$ satisfying $\left|m_{ij}(t;\bar{\theta })-m_{ij}(t)|<|m_{ij}(t;\tilde{\theta })-m_{ij}(t;\bar{\theta })\right|$ for every *i* and *j*. Rewriting $m_{ij}(t;\tilde{\theta })-m_{ij}(t)={\left(\tilde{\beta }-\beta_{0}\right)}^{\text{T}}X_{ij}(t)+({\tilde{\eta }}_{0j}-\eta_{0j})(t)$, where ${\tilde{\eta }}_{0j}(t)={\tilde{\xi }}^{\text{T}}B_{j}(t)$, and letting $\rho_{ij}(t)=p_{ij}(t)\{1-p_{ij}(t)\}$, one can show that both

$$n^{-1}\sum_{i=1}^{n}\sum_{j=1}^{J}\int_{0}^{\tau }\{Z_{ij}(t)-{\bar{z}}_{j}(t;\beta_{0})\}\rho_{ij}(t){\left(\tilde{\beta }-\beta_{0}\right)}^{\text{T}}X_{ij}(t)\{1-R_{i}(t)\}\;\text{d}N_{i\cdot }(t)$$

and

$$n^{-1}\sum_{i=1}^{n}\sum_{j=1}^{J}\int_{0}^{\tau }\{Z_{ij}(t)-{\bar{z}}_{j}(t;\beta_{0})\}\rho_{ij}(t)({\tilde{\eta }}_{0j}-\eta_{0j})(t)\{1-R_{i}(t)\}\;\text{d}N_{i\cdot }(t)$$

are $o_{p}(n^{-1/2})$ when the number of interior knots follows Condition (g) since $\tilde{\beta }-\beta_{0}=o_{p}(n^{-1/2})$ and $\int_{0}^{\tau }\left({\tilde{\eta }}_{0j}-\eta_{0j})(t)dt=o_{p}(n^{-1/2}\right)$, as derived by Wang et al.^11^ Hence

$$n^{-1/2}U_{2}^{r}(\beta_{0})=\Gamma (\beta_{0},\theta_{0})h{\left(\theta_{0}\right)}^{-1}n^{-1/2}\sum_{i=1}^{n}\sum_{j=1}^{J}U_{ij}(\theta_{0})+o_{p}(1),$$

where $\Gamma (\beta_{0},\theta_{0})=\underset{n\to \infty }{\lim}\hat{\Gamma }(\beta_{0},\theta_{0})$ and $h(\theta_{0})=\underset{n\to \infty }{\lim}H(\theta_{0})$.

Using conventional theories for empirical processes, one can also show that

$$n^{-1/2}U_{1}^{r}(\beta_{0})=n^{-1/2}\sum_{i=1}^{n}\sum_{j=1}^{J}\int_{0}^{\tau }\{Z_{ij}(t)-{\bar{z}}_{j}(t;\beta_{0})\}dM_{ij}^{r}(t;\beta_{0})+o_{p}(1)$$

Accordingly, one has $n^{-1/2}U^{r}(\beta_{0})=n^{-1/2}\sum_{i=1}^{n}\Psi_{i}(\beta_{0},\theta_{0})+o_{p}(1)$, where

$$\Psi_{i}(\beta ,\theta )=\sum_{j=1}^{J}\int_{0}^{\tau }\{Z_{ij}(t)-{\bar{z}}_{j}(t;\beta )\}\;\text{d}M_{ij}^{r}(t;\beta )+\Gamma (\beta ,\theta )h{\left(\theta \right)}^{-1}\sum_{j=1}^{J}U_{ij}(\theta )$$

The rest of the proof follows the central limit theorem.

### A.2. Proof of theorem 2

First, we let ${\hat{\omega }}_{j}(t)={\hat{\mu }}_{0j}^{r}(t;{\hat{\beta }}^{r},\tilde{\theta })-\mu_{0j}(t)$ and express $n^{1/2}{\hat{\omega }}_{j}(t)$ as $n^{1/2}{\hat{\omega }}_{j}(t)=n^{1/2}{\hat{\omega }}_{j}^{\left(1\right)}(t)+n^{1/2}{\hat{\omega }}_{j}^{\left(2\right)}(t),$ where ${\hat{\omega }}_{j}^{\left(1\right)}(t)={\hat{\mu }}_{0j}^{r}(t;{\hat{\beta }}^{r},\tilde{\theta })-{\hat{\mu }}_{0j}^{r}(t;\beta_{0},\theta_{0})$ and ${\hat{\omega }}_{j}^{\left(2\right)}(t)={\hat{\mu }}_{0j}^{r}(t;\beta_{0},\theta_{0})-\mu_{0j}(t)$. Furthermore, we let ${\hat{\omega }}_{j}^{\left(1\right)}(t)={\hat{\omega }}_{j1}^{\left(1\right)}(t)+{\hat{\omega }}_{j2}^{\left(1\right)}(t)$, where

$${\hat{\omega }}_{j1}^{\left(1\right)}(t)=n^{-1}\sum_{i=1}^{n}\int_{0}^{t}\{S_{j}^{\left(0\right)}(s;{\hat{\beta }}^{r}{)}^{-1}-S_{j}^{\left(0\right)}{\left(s;\beta_{0}\right)}^{-1}\}\;\text{d}N_{ij}^{r}(s;\tilde{\theta })$$

and

$${\hat{\omega }}_{j2}^{\left(1\right)}(t)=n^{-1}\sum_{i=1}^{n}\int_{0}^{t}S_{j}^{\left(0\right)}{\left(s;\beta_{0}\right)}^{-1}\{\text{d}N_{ij}^{r}(s;\tilde{\theta })-\text{d}N_{ij}^{r}(s;\theta_{0})\}$$

Recall that $A_{j}(t;\beta ,\theta )=-\int_{0}^{t}{\bar{Z}}_{j}(s;\beta )d{\hat{\mu }}_{0j}(s;\beta ,\theta )$. By Taylor expansion, weak law of large numbers, and consistency of $\tilde{\theta }$, one can claim that ${\hat{\omega }}_{j1}^{\left(1\right)}(t)=A_{j}{\left(t;\beta_{0},\theta_{0}\right)}^{\text{T}}({\hat{\beta }}^{r}-\beta_{0})+o_{p}(1)$ because $\partial {\hat{\omega }}_{j1}^{\left(1\right)}(t)/\partial \beta =A_{j}(t;\beta ,\theta_{0})+o_{p}(1)$, since $\underset{n\to \infty }{\lim}n^{-1}\sum_{i=1}^{n}dN_{ij}^{r}(s;\tilde{\theta })=d\mu_{0j}(t)s_{j}^{\left(0\right)}(t;\beta_{0})$ and $\underset{n\to \infty }{\lim}S_{j}^{\left(d\right)}(t;\beta_{0})=s_{j}^{\left(d\right)}(t;\beta_{0})$ for *d *=* *0, 1 uniformly in t∈[0,τ]. Under Conditions (a)–(g), one can show that $\underset{t\in [0,\tau ]}{\sup}|{\hat{\omega }}_{j1}^{\left(1\right)}(t)|$ converges to 0 since $A_{j}(t;\beta_{0},\theta_{0})$ is bounded when n→∞ and ${\hat{\beta }}^{r}$ is consistent for *β*_0_. Similarly, one can write ${\hat{\omega }}_{j2}^{\left(1\right)}(t)=n^{-1}\sum_{i=1}^{n}\int_{0}^{t}S_{j}^{\left(0\right)}{\left(s;\beta_{0}\right)}^{-1}\{p_{ij}(t;\tilde{\theta })-p_{ij}(t;\theta_{0})\}\{1-R_{i}(s)\}dN_{i\cdot }(s)$. By writing $p_{ij}(t;\tilde{\theta })-p_{ij}(t;\theta_{0})=\rho_{ij}(t;\bar{\theta })\{m_{ij}(t;\tilde{\theta })-m_{ij}(t;\theta_{0})\}$, one can show that $\underset{t\in [0,\tau ]}{\sup}|{\hat{\omega }}_{j2}^{\left(1\right)}(t)|$ converges to 0 since ${\hat{\omega }}_{j2}^{\left(1\right)}(t)=D_{j}{\left(t;\beta_{0},\theta_{0}\right)}^{\text{T}}(\tilde{\theta }-\theta )+o_{p}(1)$, consistency of $\tilde{\theta }$, and the fact that $D_{j}(t;\beta_{0},\theta_{0})$ is bounded when n→∞. Along with the uniform consistency of ${\hat{\omega }}_{j}^{\left(2\right)}(t)$, the uniform consistency of ${\hat{\omega }}_{j}(t)$ is proved.

By consistency of $\tilde{\theta }$ and ${\hat{\mu }}_{0j}(s;\beta_{0},\tilde{\theta })$ in s∈(0,t], one can claim that $A_{j}(t;\beta_{0},\tilde{\theta })$ converges in probability to $a_{j}(t;\beta_{0})=-\int_{0}^{t}{\bar{z}}_{j}(s;\beta_{0})\;\text{d}\mu_{0j}(s)$. To prove the large sample normality, one can show that $n^{1/2}{\hat{\omega }}_{j1}^{\left(1\right)}(t)=a_{j}{\left(t;\beta_{0}\right)}^{\text{T}}\Omega {\left(\beta_{0}\right)}^{-1}n^{-1/2}\sum_{i=1}^{n}\Psi_{i}(\beta_{0},\theta_{0})+o_{p}(1)$, and $n^{1/2}{\hat{\omega }}_{j2}^{\left(1\right)}(t)=d_{j}(t;\beta_{0})h{\left(\theta_{0}\right)}^{-1}n^{-1/2}\sum_{i=1}^{n}\sum_{j=1}^{J}U_{ij}(\theta_{0})+o_{p}(1)$, where

$$d_{j}(t;\beta_{0})=\underset{n\to \infty }{\lim}n^{-1}\sum_{i=1}^{n}\int_{0}^{t}s_{j}^{\left(0\right)}{\left(s;\beta_{0}\right)}^{-1}\rho_{ij}(s){\tilde{X}}_{ij}(s)\{1-R_{i}(s)\}\text{d}N_{i\cdot }(s)$$

Furthermore, one can write $n^{1/2}{\hat{\omega }}_{j}^{\left(2\right)}(t)=n^{-1/2}\sum_{i=1}^{n}\int_{0}^{t}s_{j}^{\left(0\right)}{\left(s;\beta_{0}\right)}^{-1}dM_{ij}^{r}(s;\beta_{0},\theta_{0})+o_{p}(1)$, where

$$dM_{ij}^{r}(t;\beta_{0},\theta_{0})=dN_{ij}^{r}(t;\theta )-Y_{i}(t)\exp\{\beta^{T}Z_{ij}(t)\}d\mu_{0j}(t)$$

Accordingly, the process $n^{1/2}{\hat{\omega }}_{j}(t)$ can be written as $n^{1/2}{\hat{\omega }}_{j}(t)=n^{1/2}{\hat{\omega }}_{j1}^{\left(1\right)}(t)+n^{1/2}{\hat{\omega }}_{j2}^{\left(1\right)}(t)+n^{1/2}{\hat{\omega }}_{j}^{\left(2\right)}(t)$, which can be expressed as $n^{-1/2}\sum_{i=1}^{n}\phi_{ij}(t;\beta_{0},\theta_{0})+o_{p}(1)$, where

$$\begin{array}{l} \phi_{ij}(t;\beta_{0},\theta_{0})=a_{j}{\left(t;\beta_{0}\right)}^{\text{T}}\Omega {\left(\beta_{0}\right)}^{-1}\Psi_{i}(\beta_{0},\theta_{0})\\ \;+d_{j}(t;\beta_{0})h(\theta_{0})-1\sum_{j=1}^{J}U_{ij}(\theta_{0})+\int_{0}^{t}s_{j}^{\left(0\right)}(s;\beta_{0})-1dM_{ij}^{r}(s;\beta_{0},\theta_{0}) \end{array}$$

One can see that $n^{-1/2}\sum_{i=1}^{n}\phi_{ij}(t;\beta_{0},\theta_{0})$ is a normalized sum of independent and identically distributed random variables and, by the central limit theory, converges to a multivariate normal distribution with mean zero and covariance $V_{j}(s,t)=E\{\phi_{1j}(s;\beta_{0},\theta_{0})\phi_{1j}(t;\beta_{0},\theta_{0})\}$ given finitely many s,t∈[0,τ]. Since $\phi_{1j}(t;\beta_{0},\theta_{0})$ is monotone in *t*, $n^{1/2}{\hat{\omega }}_{j}(t)$ is tight and hence converges weakly to a Gaussian process by the functional central limit theorem.
