# Supplementary material for: Semiparametric estimation of the proportional rates model for recurrent events data with missing event category
Source: Stat Methods Med Res. 2021 Jun 18;30(7):1624–39. doi: 10.1177/09622802211023975 (PMC8411467; doi:10.1177/09622802211023975)
Supplement: sj-pdf-1-smm-10.1177_09622802211023975 - Supplemental material for Semiparametric estimation of the proportional rates model for recurrent events data with missing event category [file sj-pdf-1-smm-10.1177_09622802211023975.pdf]

Supplementary Materials for "Semiparametric estimation of the proportional rates model for recurrent events data with missing event category"

by Feng-Chang Lin, Jianwen Cai, Jason P. Fine, Elisabeth P. Dellon, Charles R. Esther Jr.

1 Additional Simulations for a Misspecified Model

In this section, we present the estimation results under a misspecified model to show the robustness of our methodology. The data were generated by two intensity functions

$$\lambda_{i1}(t) = G_i r_{01} \exp(\beta_1 \tilde{Z}_i + \beta_3 \tilde{W}_i),$$

and

$$\lambda_{i2}(t) = G_i r_{02} h_0(t) \exp(\beta_2 \tilde{Z}_i + \beta_4 \tilde{W}_i),$$

for type 1 and 2 recurrent events, respectively, where all of the parameters and variables were set up the same as the scenario 2 in the simulation section of our main manuscript except β_3, β_4, and $\tilde{W}_i$. Here, we intend to create the model misspecification by a rate function

$$E\{dN_{ij}^*(t)|Z_{ij}(t)\} = \exp(\beta_0^{\mathrm{T}} Z_{ij}) d\mu_{0j}(t), \tag{1}$$

for $j = 1, 2$, where $\beta_0 = (\beta_1, \beta_2)^{\mathrm{T}}$, $Z_{ij} = (\tilde{Z}_i, 0)$ if $j = 1$, and $Z_{ij} = (0, \tilde{Z}_i)$ if $j = 2$. The fitting rate function missed the important covariate $\tilde{W}_i$ that was generated uniformly between 0 and 1. We let $\beta_3 = -\log(2)$ and $\beta_4 = 0$. The simulation was repeated 500 times under $n = 200, 400$. The same as Table 2 in our main paper, we report bias, empirical standard deviation (σ), average of the standard error estimates ($\bar{\sigma}$), coverage probability ($\mathcal{C}_p$) based on the 95% confidence interval for β_1, and empirical rejection rate ($\mathcal{C}_0$) when testing the null hypothesis $\beta_2 = 0$ by a Wald-type test.

Table S1 shows the simulation result. One can see the misspecification of the rate model (1) that misses the critical covariate $\tilde{W}_i$ affects little the performance of our proposed estimator. The estimates are approximately unbiased and the approximation of the asymptotic variance performed well under such misspecification. The coverage probability is around the 0.95 nominal level, even when the censoring rate is high, and the empirical rejection rate is close to the 0.05 type-I error probability.

We also purposely use a misspecified weighted estimating equations method that ignores $\tilde{W}_i$. We intend to show that the our estimator is more efficient than the weighted estimating equations method when the model is misspecified. The simulation results are shown in Table S2. The same as the Table 1 in our main paper, we report the bias, empirical standard error of the estimator (σ_1), and mean square error ratios comparing to the estimators assuming no missing event category and using weighted estimating equations.

Table S2 shows that, when the model is misspecified by the weighted estimating equations method, the estimator $\hat{\beta}_1^w$ remains approximately unbiased when the sample size n is large. However, the variation is relatively large, compared to our estimator, especially when the censoring rate is high. Our estimator can improve the efficiency up to 20% when the censoring rate is at 40%.

2 Additional Simulations for Time-Varying Covariate Models

In this section, we present the simulation results under a model with a time-varying covariate to show that our estimation method can also handle time-varying covariates. We generated the data by the following intensity functions

$$\lambda_{i1}(t) = G_i r_{01} \exp\{\beta_1 \tilde{Z}_i + \beta_3 \tilde{W}_i I(t \geq 2)\},$$

and

$$\lambda_{i2}(t) = G_i r_{02} h_0(t) \exp\{\beta_2 \tilde{Z}_i + \beta_4 \tilde{W}_i I(t \geq 2)\},$$

where $\tilde{W}_i$ is a binary random variable from a Bernoulli distribution with probability 0.5, and $I(\cdot)$ is an indicator function that indicates the time-varying effect of $\tilde{W}_i$ on the intensity. All of the parameters and variables were set up the same as the scenario 2 in the simulation section of our main manuscript except β_3, β_4, and $\tilde{W}_i$. We let $\beta_3 = \log(0.8)$ and $\beta_4 = 0$. The negative value of β_3 suggests that $\tilde{W}_i$ decreases the intensity of type 1 events when t

Table S1: Simulation results of parameter estimation under a misspecified model

					$\hat{\beta}_1^r$				$\hat{\beta}_2^r$			
(β_1, β_2)	α	ϵ_z	$\mathcal{M}_p$	n	bias	σ_1	$\bar{\sigma}_1$	$\mathcal{C}_p$	bias	σ_2	$\bar{\sigma}_2$	$\mathcal{C}_0$
(0.69,0)	0	0	20%	200	0.001	0.286	0.279	0.940	0.002	0.155	0.150	0.056
				400	0.001	0.204	0.196	0.936	-0.006	0.110	0.106	0.056
			30%	200	0.000	0.299	0.291	0.940	0.003	0.159	0.153	0.054
				400	0.000	0.214	0.205	0.944	-0.005	0.113	0.108	0.056
			40%	200	-0.005	0.317	0.303	0.938	0.005	0.160	0.155	0.066
				400	0.000	0.222	0.213	0.942	-0.005	0.116	0.109	0.054
		0.69	20%	200	0.002	0.286	0.278	0.954	0.002	0.156	0.151	0.052
				400	0.001	0.202	0.195	0.942	-0.006	0.111	0.106	0.060
			30%	200	-0.005	0.296	0.287	0.940	0.004	0.159	0.153	0.058
				400	-0.002	0.209	0.201	0.938	-0.005	0.114	0.108	0.054
			40%	200	0.001	0.309	0.298	0.946	0.001	0.159	0.156	0.044
				400	0.001	0.222	0.209	0.938	-0.007	0.114	0.110	0.054
	0.5	0	20%	200	-0.011	0.311	0.304	0.942	-0.004	0.196	0.188	0.060
				400	-0.017	0.217	0.213	0.940	0.003	0.141	0.133	0.062
			30%	200	-0.005	0.322	0.316	0.950	-0.005	0.199	0.190	0.064
				400	-0.011	0.226	0.221	0.942	0.001	0.144	0.135	0.060
			40%	200	-0.002	0.336	0.326	0.936	-0.006	0.203	0.192	0.060
				400	-0.009	0.235	0.228	0.942	0.000	0.146	0.136	0.066
		0.69	20%	200	-0.013	0.306	0.302	0.942	-0.003	0.195	0.188	0.054
				400	-0.018	0.214	0.211	0.934	0.003	0.141	0.134	0.058
			30%	200	-0.010	0.321	0.311	0.932	-0.005	0.198	0.190	0.056
				400	-0.014	0.225	0.218	0.936	0.001	0.144	0.135	0.056
			40%	200	-0.005	0.335	0.324	0.942	-0.007	0.201	0.194	0.066
				400	-0.009	0.236	0.224	0.940	-0.001	0.144	0.136	0.074

Table S2: Simulation results based on the misspecified model

					bias			σ_1			$ratio$	
β_1	α	n	ϵ_z	$\mathcal{M}_p$	$\hat{\beta}_1^n$	$\hat{\beta}_1^r$	$\hat{\beta}_1^w$	$\hat{\beta}_1^n$	$\hat{\beta}_1^r$	$\hat{\beta}_1^w$	e_r^n	e_r^w
0.69	0	200	0	20%	0.008	0.001	-0.001	0.271	0.286	0.294	0.90	1.06
				30%		0.000	-0.001		0.299	0.312	0.82	1.09
				40%		-0.005	-0.001		0.317	0.343	0.73	1.17
			0.69	20%		0.002	0.001		0.286	0.292	0.89	1.04
				30%		-0.005	-0.007		0.296	0.309	0.84	1.09
				40%		0.001	-0.009		0.309	0.324	0.77	1.10
		400	0	20%	0.002	0.001	0.002	0.192	0.204	0.212	0.88	1.07
				30%		0.000	-0.002		0.214	0.222	0.81	1.08
				40%		0.000	0.002		0.222	0.237	0.75	1.14
			0.69	20%		0.001	0.001		0.202	0.208	0.90	1.06
				30%		-0.002	-0.001		0.209	0.220	0.84	1.10
				40%		0.001	-0.006		0.222	0.238	0.75	1.15
	0.5	200	0	20%	-0.010	-0.011	-0.012	0.289	0.311	0.322	0.86	1.07
				30%		-0.005	0.001		0.322	0.345	0.80	1.15
				40%		-0.002	0.006		0.336	0.367	0.74	1.20
			0.69	20%		-0.013	-0.014		0.306	0.316	0.89	1.07
				30%		-0.010	-0.009		0.321	0.340	0.81	1.12
				40%		-0.005	-0.003		0.335	0.363	0.75	1.17
		400	0	20%	-0.013	-0.017	-0.016	0.206	0.217	0.223	0.90	1.06
				30%		-0.011	-0.008		0.226	0.238	0.84	1.11
				40%		-0.009	-0.001		0.235	0.250	0.77	1.13
			0.69	20%		-0.018	-0.018		0.214	0.221	0.93	1.07
				30%		-0.014	-0.013		0.225	0.235	0.84	1.08
				40%		-0.009	-0.005		0.236	0.248	0.76	1.10

is larger than or equal to 2. We fit the data by rate functions

$$E\{dN_{ij}^*(t)|Z_{ij}, W_{ij}\} = \exp\{\beta_{10}^{\mathrm{T}} Z_{ij} + \beta_{20}^{\mathrm{T}} W_{ij} I(t \geq 2)\} d\mu_{0j}(t), \tag{2}$$

for $j = 1, 2$, where $\beta_{10} = (\beta_1, \beta_2)^{\mathrm{T}}$, $Z_{i1} = (\tilde{Z}_i, 0)^{\mathrm{T}}$, and $Z_{i2} = (0, \tilde{Z}_i)^{\mathrm{T}}$; $\beta_{20} = (\beta_3, \beta_4)^{\mathrm{T}}$, $W_{i1} = (\tilde{W}_i, 0)^{\mathrm{T}}$, and $W_{i2} = (0, \tilde{W}_i)^{\mathrm{T}}$. Model (2) is considered as a time-varying covariate model since the covariate $W_{ij} I(t \geq 2)$ changes by time.

Table S3 shows the simulation result based on 500 repeated samples under sample sizes $n = 200, 400$. We reported the same metrics in Table S1 for β_1 and β_3, where β_3 is the coefficient of the time-varying covariate. Form Table S3, one can see that our estimator behave similarly when the covariate is time-varying. Both point and variance estimation perform well, resulting around 0.95 coverage probability. The simulation result demonstrates our estimator can work under a time-varying covariate model.

3 Assessment of Missing at Random Assumption

The missing at random (MAR) assumption (Little and Rubin, 2002) can be assessed by exploring whether the likelihood of missingness depends covariates. One can assume the probability of having a missing category follows a logistic regression model

$$1 - \pi_i(t) = [1 + \exp\{-\epsilon' z_i(t)\}]^{-1},$$

where $z_i(t) = (1, t, N_{i\cdot}(t-), Z_i)'$ and ϵ is a column vector of corresponding coefficients of $z_i(t)$. To assess the MAR assumption in the Cystic Fibrosis Foundation Patient Registry (CFFPR) data, we include all of the covariates in Table 3 of the main paper for Z_i and fit the logistic regression model above. Table S4 shows the coefficient estimation in odds ratio, its 95% confidence interval (CI), and Wald-type test p-value. The result indicates that the missingness is significantly associated with age, number of previous events, diagnostic method, and medication use. The overall testing for $\epsilon = 0$ is significant (p-value<0.001), which indicates the missingness depends on the covariates.

4 Sensitivity Analysis when Missing at Random Assumption is Violated

The missing at random (MAR) assumption stands if $\kappa_j(t|Z_{ij}) = 0$ in model (5) of the main paper. To check the robustness of our analysis for the Cystic Fibrosis Foundation

Table S3: Simulation results of parameter estimation under a type-varying covariates model

					$\hat{\beta}_1^r$				$\hat{\beta}_3^r$			
(β_1, β_3)	α	ϵ_z	$\mathcal{M}_p$	n	bias	σ_1	$\bar{\sigma}_1$	$\mathcal{C}_p$	bias	σ_2	$\bar{\sigma}_2$	$\mathcal{C}_p$
(0.69,-0.22)	0	0	20%	200	0.022	0.240	0.240	0.954	-0.006	0.414	0.396	0.946
				400	0.014	0.162	0.168	0.952	0.015	0.272	0.273	0.960
			30%	200	0.027	0.245	0.248	0.946	-0.003	0.425	0.406	0.952
				400	0.019	0.164	0.173	0.964	0.014	0.284	0.280	0.956
			40%	200	0.028	0.260	0.260	0.954	-0.001	0.447	0.423	0.960
				400	0.021	0.174	0.182	0.946	0.014	0.299	0.291	0.946
		0.69	20%	200	0.025	0.239	0.238	0.946	-0.007	0.415	0.398	0.954
				400	0.014	0.160	0.166	0.954	0.010	0.275	0.274	0.960
			30%	200	0.023	0.242	0.246	0.950	-0.001	0.424	0.409	0.956
				400	0.016	0.163	0.172	0.964	0.017	0.286	0.282	0.952
			40%	200	0.026	0.251	0.256	0.952	0.001	0.444	0.423	0.954
				400	0.017	0.173	0.179	0.954	0.018	0.304	0.292	0.948
	0.5	0	20%	200	0.025	0.266	0.265	0.948	0.003	0.452	0.429	0.940
				400	0.011	0.197	0.186	0.932	0.006	0.312	0.297	0.946
			30%	200	0.026	0.280	0.274	0.948	0.004	0.470	0.445	0.946
				400	0.014	0.205	0.192	0.928	0.007	0.321	0.306	0.946
			40%	200	0.030	0.293	0.283	0.942	0.009	0.482	0.458	0.940
				400	0.013	0.209	0.198	0.936	0.010	0.323	0.314	0.934
		0.69	20%	200	0.025	0.262	0.263	0.952	0.003	0.453	0.431	0.946
				400	0.011	0.199	0.185	0.920	0.006	0.312	0.297	0.948
			30%	200	0.024	0.272	0.270	0.938	0.004	0.458	0.444	0.946
				400	0.013	0.202	0.190	0.926	0.006	0.318	0.306	0.946
			40%	200	0.022	0.289	0.279	0.940	0.008	0.509	0.459	0.934
				400	0.007	0.206	0.197	0.938	0.007	0.337	0.316	0.942

Table S4: Regression analysis of the likelihood of missingness in the CF registry data

Covariates	$\exp(\epsilon)$	95% CI	p-value
Age	1.02	1.00, 1.05	0.016
Number of previous events	0.94	0.92, 0.96	<0.001
Female	0.94	0.85, 1.04	0.211
Genotype F508del			
Homozygous	1.00	-	
Heterozygous	0.99	0.89, 1.10	0.870
Neither or unknown	1.04	0.87, 1.24	0.658
Diagnostic method			
Newborn screening	1.00	-	
Meconium ileus	0.81	0.70, 0.92	0.002
Family history	0.68	0.49, 0.95	0.026
Symptom	0.77	0.68, 0.87	<0.001
Medication	1.26	1.15, 1.37	<0.001

Patient Registry (CFFPR) data, we assigned different values of $\kappa_j(t|Z_{ij})$ in the model (5) and examined whether the coefficient estimation of the proportional rates model is sensitive to different values of $\kappa_j(t|Z_{ij})$. Here, we assigned constant values for $\kappa_1(t|Z_{ij})$ and $\kappa_2(t|Z_{ij})$ that are independent of Z_{ij}. Figures S1 and S2 show the estimation results under different values of κ_1 and κ_2 when randomly choosing 10% of the patient's data for the sensitivity analysis. One can see the rate ratio estimation of the sex effect of female relative to male in nonmucoid PA infection in the solid lines of Figure S1 varies little under different combinations of κ_1 and κ_2. Our proposed estimates are shown in a triangle when $\kappa_1 = \kappa_2 = 0$. Although the 95% confidence intervals are not shown, they also have little difference based on the proposed variance estimation. A similar result can be seen in Figure S2, where the point estimation are shown for the effect of the heterozygous relative to homozygous F508del genotype in nonmucoid PA infection. The result shows that our proposed estimators for the CFFPR data are quite robust to various values we considered for κ_1 and κ_2.

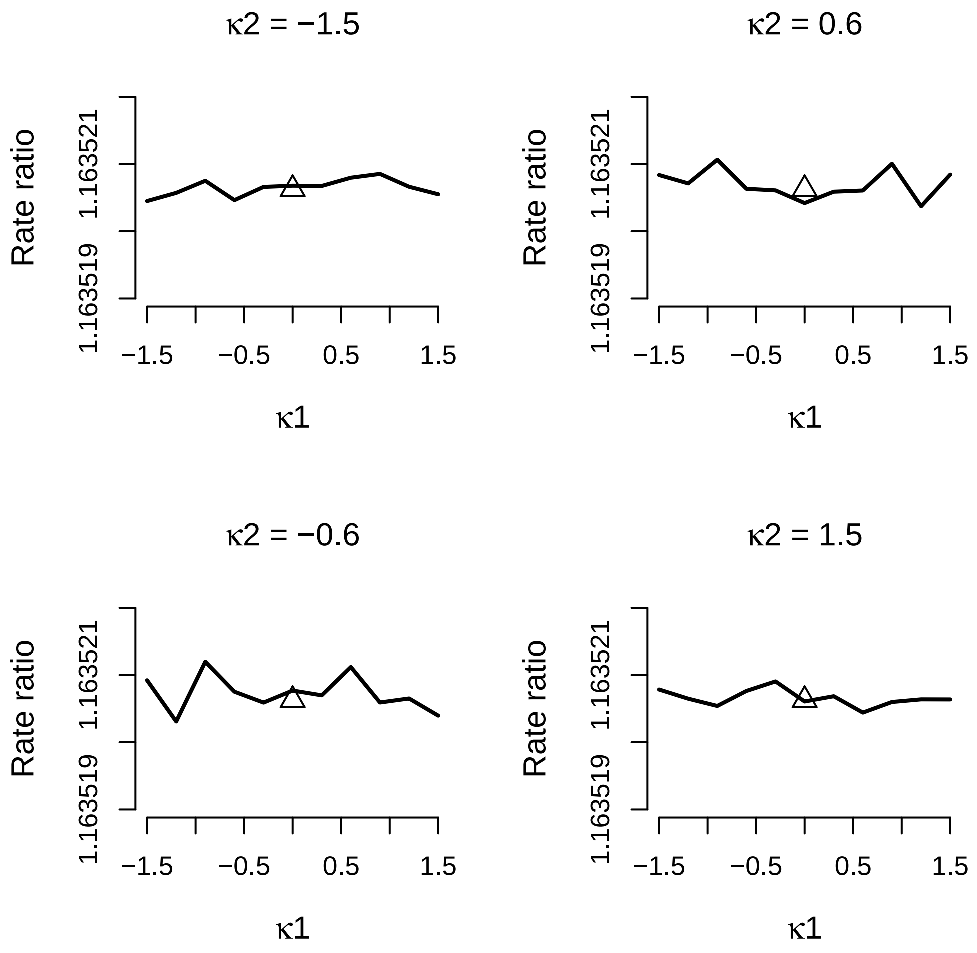

Figure S1: Sensitivity analysis for the effect of female compared to male in nonmucoid PA infection under four values of κ_2 and a sequence of κ_1 values from -1.5 to 1.5

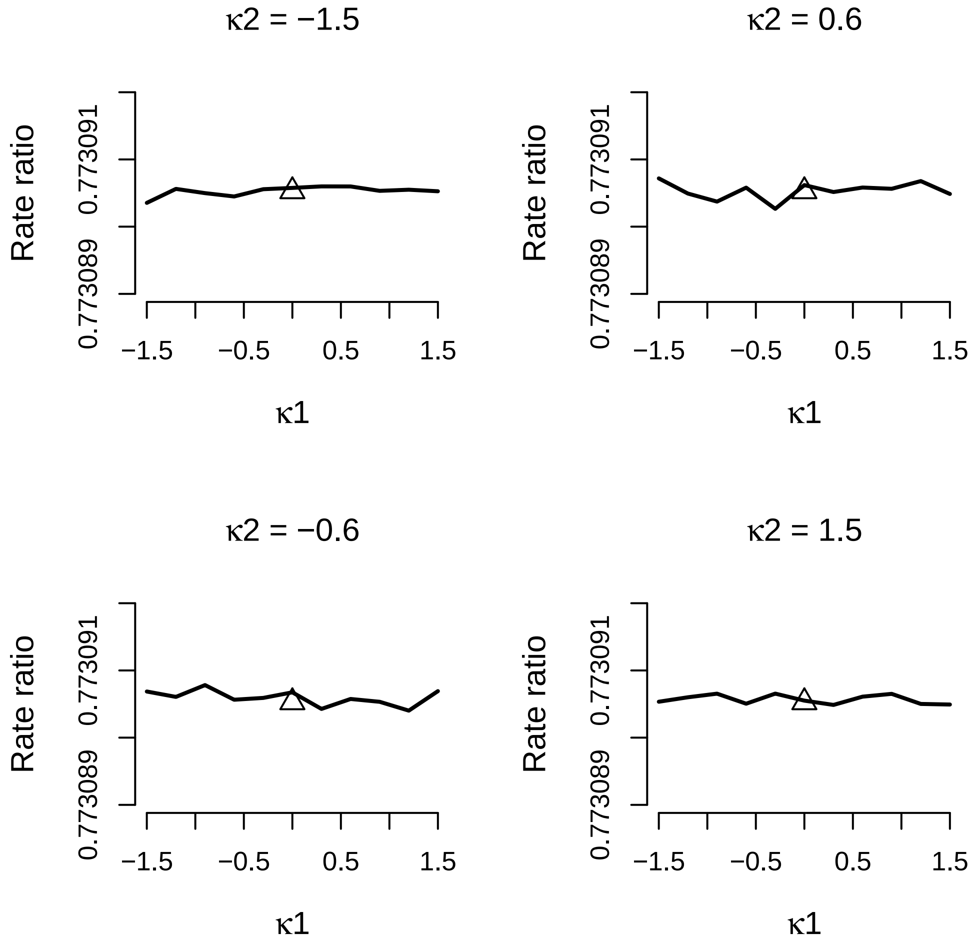

Figure S2: Sensitivity analysis for the effect of heterozygous compared to homozygous F508del genotype in nonmucoid PA infection under four values of κ_2 and a sequence of κ_1 values from -1.5 to 1.5
